# Promoting positive beliefs toward research evidence: results from a utility-value intervention study with pre-service teachers

**DOI:** 10.3389/fpsyg.2025.1391931

**Published:** 2025-08-06

**Authors:** Maximilian Knogler, Ricardo Böheim, Annika Diery, Judith Harackiewicz, Tina Seidel

**Affiliations:** ^1^School of Social Sciences and Technology, Technical University of Munich (TUM), Munich, Germany; ^2^Department of Psychology, University of Wisconsin-Madison, Madison, WI, United States

**Keywords:** utility-value, teacher education, evidence-based practice, pre-service teachers, intervention

## Abstract

Pre-service teachers often question the relevance of educational evidence for professional practice. Yet, according to expectancy-value theory, the extent to which pre-service teachers consider educational evidence relevant for their teaching practice (i.e., utility-value) is a critical variable in promoting evidence-based practice in education. To further promote utility-value of research evidence among pre-service teachers, the present study investigated the added value of a brief and easy-to-implement intervention that stimulates students to reflect on the utility-value of research evidence. The sample consisted of 3^rd^ semester pre-service teachers (*N* = 61) enrolled in a semester-long course on effective teaching who were randomly assigned to two conditions. In the first condition (default course design), teacher educators used two typically applied strategies for promoting utility-value, i.e., direct communication of utility-value and application tasks, in which students can discover utility-value. In the second condition (enhanced course design), students were additionally stimulated to reflect on the utility-value in two written assignments. Their value perceptions and related variables were measured at the beginning, during and at the end of the semester. Although a mixed model MANOVA did not yield a statistically significant group-by-time interaction effect, follow-up *t*-tests revealed a substantial and significant increase in students value perceptions in the enhanced course design, but not in the default course design. Overall, this study offers some limited support for the additional value of reflective writing assignments for fostering pre-service teachers' positive beliefs toward research evidence in education.

## Introduction

Following the movement toward evidence-based practice in education, teaching is regarded as a profession that requires teachers to act and argue based on research evidence (e.g., Bauer and Prenzel, 2012; European Commission [EC], 2007; U.S. Department of Education, Office of Elementary and Secondary Education, 2002; Ferguson, 2021). Originating from the medical sciences (Sackett et al., 1996), evidence-based practice in education refers to decision-making processes in professional practice that are informed by the best available evidence. As a complementary resource, scientific evidence can have multiple functions by informing teachers' thinking, their lesson design, professional evaluation, reflection and discourse (Cain et al., 2019; Stark, 2017). The term “evidence” refers to scientific information and knowledge generated systematically through scientific methods as opposed to personal experiential and anecdotal information (e.g., Dekker and Meeter, 2022). Prior research has demonstrated that increasing knowledge and use of scientific evidence can profit teachers as professionals and in turn improve instructional quality and student learning as the major goal of the teaching profession (e.g., König and Pflanzl, 2016; Ulferts, 2019; Voss et al., 2011, 2022).

Despite the increasing availability of reliable and relevant research evidence to inform teaching practice (e.g., Hedges, 2018; Knogler et al., 2022; Renkl, 2022), recent research has shown that both pre-service and in-service teachers rarely consult and act on scientific information when facing professional tasks (Brown and Rogers, 2015; Dagenais et al., 2012; Franke and Wecker, 2019; Ferguson and Bråten, 2022; Hetmanek et al., 2015; Neuweg, 2011; Patry, 2019; Thomm et al., 2021b; Trempler et al., 2015; Wenglein et al., 2015). This is not surprising: the implementation of evidence-based practice has been found to be a complex endeavor across various professional domains and fields of practice. From a psychological perspective, evidence-based practice involves a range of abilities, sufficient motivation, and opportunities to practice (see Rousseau and Gunia, 2016). This is also true for the teaching profession. A recent literature review identified a lack of teachers' skills and their skeptical beliefs and negative attitudes as some of the major barriers for teachers' utilization of scientific evidence (see van Schaik et al., 2018). Thus, efforts to increase the implementation of evidence-based practice on the individual level may have at least two leverage points. First, interventions can focus on developing teachers' skills in selecting, understanding and applying evidence to practical contexts (e.g., Cain, 2015; Engelmann et al., 2022; Furinghetti and Pehkonen, 2002; Wagner et al., 2018; Wenglein et al., 2015). And second, interventions can target teachers' skeptical beliefs e.g., about the validity, utility and applicability of research evidence in classrooms (e.g., Kiemer and Kollar, 2021; Rochnia and Gräsel, 2022; Zeeb et al., 2019).

With this research, we focus on the latter: teachers' beliefs. Beliefs and attitudes are critical as they can act as facilitators or barriers to activities related to evidence-based practice (Stark, 2017). A critical variable in this context is the extent to which (pre-service) teachers consider research evidence useful for their teaching practice (i.e., utility-value; Wigfield and Eccles, 2020; Watt and Richardson, 2015). Recent research has shown that targeted inventions have the potential to promote utility-value beliefs in different contexts (Harackiewicz and Priniski, 2018). We believe that initial teacher education at university is a critical window for targeted intervention and with the current study we seek to leverage this potential in the context of teacher education. In this study, we experimentally test the added value of reflective writing exercises for promoting pre-service teachers' perceptions of utility value in a pre-registered field experiment. Overall, we aim at generating findings which can inform effective course design in teacher education for promoting positive beliefs toward research evidence.

### The context: pre-service teachers and university-based teacher education

Most teaching careers require a university education and degree (Bauer and Prenzel, 2012). Universities are institutions which typically combine research and teaching in university-based teacher education (Ferguson, 2021). As such, teacher education programs at university seek to integrate theoretical knowledge and empirical findings with specific contexts for application and opportunities for practical experiences (Darling-Hammond, 2017). Besides, university-based teacher educators are often trained and involved in research activities and support evidence-based practice in teacher education (Diery et al., 2021; Canning et al., 2018). Thus, university-based teacher education can be understood as an epistemic system—that is, a structured environment in which knowledge is produced, communicated, and critically evaluated. Within this system, preservice teachers‘ acquisition of knowledge, appreciation of the utility of educational research, and experiences with applying it can be actively fostered by teacher educators (Greene, 2016; Ferguson, 2021). In this context, we understand evidence as empirically grounded knowledge generated through educational research, which can inform teaching and professional decision-making (e.g., Dekker and Meeter, 2022). University-based teacher education contributes to evidence-based practice both by conveying such research-based content (i.e., research as knowledge to be learned) and by fostering preservice teachers' competencies to critically engage with and apply research findings in pedagogically meaningful ways (i.e., research as a professional practice).

Moreover, findings demonstrate that once teachers have left university, they not only face increasing difficulties with accessing scientific evidence, but also that current school organization and culture do not provide much time, support and incentives to find, read and apply evidence in the classroom (van Schaik et al., 2018; Helmsley-Brown and Sharp, 2003). Hence, university-based teacher education represents a critical window and learning environment for future teachers shaping their beliefs toward evidence (Greene, 2016). University courses may thus be the best place to start promoting pre-service teachers' positive beliefs and attitudes about the importance of evidence-based practice. To optimally harness the opportunity, research on course designs which best promote positive beliefs is crucial.

### The target: pre-service teachers' beliefs toward educational research evidence

Teacher beliefs are suppositions (subjective views about the self and the world that are thought to be true) held by (pre-service) teachers that have relevance for their professional development and practice (Fives and Gill, 2015). Previous research has identified a plethora of teacher beliefs with regard to teaching, to student learning and to sources of teaching knowledge (Fives and Buehl, 2008). With regard to evidence-based practice, epistemological beliefs about the nature and complexity of knowledge, the validity and trustworthiness of science and the utility of science are most relevant (Hofer and Pintrich, 1997; Fives and Buehl, 2012; Schoor and Schütz, 2021). In general, these beliefs have shown to influence teachers' consideration of different sources of knowledge, how they interpret and engage with information and what they transfer to practical contexts (Pajares, 1992; Reusser and Pauli, 2014).

To specify beliefs which are critical for pre-service teachers' evidence-based practice, this research draws on the theoretical framework of expectancy-value theory. The theory posits that individuals' intentions, choices, performance and persistence related to tasks and careers are determined by their expectancies for success and subjective task value (Eccles and Wigfield, 2020). Empirical findings suggest that expectancies are more strongly related to performance, whereas subjective task value is a stronger predictor of intentions and actual choices (Bong, 2001; Durik et al., 2006; Eccles, 2011; Wigfield and Cambria, 2010). This pattern has been confirmed in other contexts (e.g., STEM education), as several studies have linked subjective task value to choice-related outcomes such as course enrollment and college major (Harackiewicz and Priniski, 2018). Given the connection between task value and choice in these contexts, it is plausible that helping students to see the value in research evidence could lead to strengthening future teachers' intentions to consider and use findings from research in order to inform their teaching (see also Fishbein and Ajzen, 2010; Zeeb and Voss, 2025).

Recent empirical research in the context of teacher education has investigated the hypothesized theoretical link between pre-service teachers' perceived utility value of educational research and different outcomes more directly related to consideration and implementation of scientific evidence in teaching. For example, Bråten and Ferguson (2015) found evidence that more positive beliefs by student teachers about the importance of formalized sources of knowledge (such as research articles or textbooks) are associated with higher motivation to learn from formal teacher training courses (see also Chan, 2003; Siegel and Daumiller, 2021; Ferguson et al., 2022). More recently, Voss (2022), corroborated these findings by demonstrating that more skeptical beliefs about the importance of education science in a sample of pre-service teachers were associated with lower engagement with research from education and less openness to scientific evidence (see also Fives and Buehl, 2012). Similarly, Kiemer and Kollar (2021) found that pre-service teachers' beliefs about the utility of educational theories and evidence were predictive for both selection and use of scientific sources when analyzing problematic classroom situations (see also Gold et al., 2024; Greisel et al., 2022). Moreover, a recent study with in-service teachers (Nägel et al., 2023) showed that teachers who reported a higher skepticism toward the relevance of scientific content for teaching practice, reported a significantly lower preference for research literature and a higher preference for non-formal sources of information. Finally, recent experimental research in teacher education (Zeeb and Voss, 2025) has confirmed that increasing utility value among pre-service teachers can foster stronger intentions to engage with educational research. Taken together, these findings demonstrate that pre-service and in-service teachers, who report higher levels of utility value, are more likely to engage with sources related to educational science. This strengthens the notion that teachers' value beliefs may matter for their endorsement and development of evidence-based practice (Ferguson et al., 2022).

Contrary to findings from empirical research and teacher education policy standards which both highlight the importance of knowledge from educational science for teaching success (Voss et al., 2014, 2022; König and Pflanzl, 2016), previous research also provides evidence that (pre-service) teachers often contest the usefulness of scientific evidence for their (future) practice (Allen, 2009; Fajet et al., 2005; Gitlin et al., 1999; van Schaik et al., 2018). Yet, these findings are nuanced. In many studies, mean values of self-reported utility ratings center around the numerical scale mean and neither demonstrate a clear negative nor a clear positive trend (Nägel et al., 2023; Thomm et al., 2021a; Voss, 2022; Kiemer and Kollar, 2021; Rochnia and Gräsel, 2022; Ferguson et al., 2022). Thus, recent research indicates that (pre-service) teachers on average acknowledge the utility value of educational science to a certain degree. However, findings also demonstrate that pre-service teachers on average see more utility-value in non-scientific sources (i.e., anecdotal and experiential information) as compared to scientific sources when confronted with classroom challenges or topics in educational psychology (Ferguson et al., 2022; Kiemer and Kollar, 2021; Menz et al., 2021). Consequently, it seems vital to create learning opportunities in university teacher training programs that support students in reflecting on their beliefs about education science and its importance for classroom teaching. Whereas the perceived utility value of evidence-based practice can decrease during school internships for pre-service teachers (Bleck and Lipowsky, 2020), Voss (2022) shows that master's level student teachers can hold more positive utility beliefs about educational science than their counterparts at the bachelor level. Thus, well-designed teacher training courses at university which target students' utility-value perceptions may make a difference.

### The intervention: designing a utility-value intervention for pre-service teacher education

Researchers have found that targeted psychological interventions can have powerful and long-lasting effects in higher education (Harackiewicz and Priniski, 2018). Specifically, utility-value interventions have been shown to be effective across various student populations and learning outcomes (e.g., Hulleman and Harackiewicz, 2020; Lazowski and Hulleman, 2016). Based on expectancy value theory, the hypothesis driving the utility-value intervention is that if students are supported in finding value in course content, this will increase their motivation to engage with this content and in turn increase their performance and/or strengthen their intention to pursue course-related activities or careers (Eccles and Wigfield, 2020). Utility value is defined as the value students perceive in a task or topic as a consequence of its usefulness for achieving short- or long-term goals. In the context of pre-service teacher training and evidence-based practice, utility-value interventions can target pre-service teachers and help them find value in educational science topics and findings for achieving goals related to their (future) teaching during internships or to their future jobs. For example, pre-service teachers might perceive utility value in educational science because they can use findings related to the effectives and implementation of different teaching strategies (Knogler et al., 2022) to improve their teaching.

Although utility-value interventions primarily focus on enhancing learners' perceptions of the utility value of course content, recent theorizing and empirical findings suggest that the psychological processes they instigate can also influence other key motivational beliefs (Hulleman and Harackiewicz, 2020). In the context of pre-service teacher training and evidence-based practice these include competence beliefs, interest, and behavioral intentions. Competence beliefs, defined as students‘ confidence in their ability to succeed in a particular task or domain, are strengthened when students perceive learning activities as personally meaningful. By connecting coursework to their personal goals, students often gain a greater sense of mastery over the material, which in turn boosts their confidence, e.g., in dealing with research evidence (Brisson et al., 2017; Canning and Harackiewicz, 2015; Hulleman and Harackiewicz, 2020; Durik and Harackiewicz, 2006). Simultaneously, utility-value interventions have been shown to increase interest—the enjoyment and intrinsic value students find in the subject. By emphasizing the personal relevance of the material, these interventions spark curiosity and engagement, transforming situational interest into a more enduring, personal interest (Hulleman et al., 2010; Harackiewicz and Knogler, 2017; Rosenzweig et al., 2020). Moreover, as students perceive greater value and interest in what they are learning, their behavioral intentions are strengthened—they become more motivated to invest effort, persist in the course, pursue future academic or career opportunities in the field. In essence, elevating perceived utility of research evidence can fosters stronger intentions to engage in related behaviors such as using evidence from educational research for lesson design etc. (Hulleman and Harackiewicz, 2020; Harackiewicz et al., 2016).

Notably, empirical evidence indicates that these effects are especially pronounced for students with low initial confidence or those from underrepresented backgrounds. Students who begin with lower competence beliefs or weaker academic performance gain the most from utility-value interventions, showing substantial improvements in their sense of competence, interest, and intent to persist (Hulleman and Harackiewicz, 2009, 2020). For example, a self-generated utility intervention significantly boosted both science course interest and grades, particularly for students with initially low success expectations (Hulleman and Harackiewicz, 2009). Similarly, in college STEM courses, utility-value interventions have helped narrow achievement gaps by improving performance and persistence, especially for first-generation and minority students—groups that often face confidence challenges—thereby reinforcing the efficacy of utility-value interventions for those in need of motivational support (Harackiewicz et al., 2016; Hulleman and Harackiewicz, 2020). In conclusion, by enhancing the perceived usefulness of academic content, utility-value interventions foster increased competence beliefs, both situational and sustained interest, and stronger intentions to engage with this content.

From the literature, we identified three different strategies to support the perception of utility-value in teacher education: (1) Direct communication of utility-value, (2) self-generated utility-value through application tasks, and (3) self-generated utility-value through reflective writing tasks (e.g., Durik and Harackiewicz, 2006; Knogler and Lewalter, 2014a,b; Hulleman and Harackiewicz, 2009). Since teacher educators at university predominantly support evidence-based practice in teaching (e.g., Diery et al., 2020, 2021), course designs in current teacher education programs may often include some of these strategies to support the perception of utility-value.

In this research, we regard direct communication of value (Strategy 1) and application tasks (Strategy 2) as default design features of teacher education courses. During courses in teacher education, educators typically directly communicate the value of research findings, e.g., by emphasizing the usefulness and importance of a certain course content through their presentations or through course material (Bauer and Prenzel, 2012). To some extent this type of direct persuasion, might convince future teachers of the usefulness of evidence from educational science (e.g., Durik and Harackiewicz, 2007). Moreover, courses in teacher education typically include tasks in which students are asked to apply course content in authentic professional contexts such as teaching scenarios and simulations (Fischer et al., 2022). By tasking students to explicitly apply course content in professionally authentic contexts, pre-service teachers may discover and experience its usefulness in addressing professional challenges, which might increase their utility-value perceptions of current research evidence (Kember et al., 2008).

For both strategies, there is currently mixed or limited evidence of effectiveness. Direct communication of utility-value yielded mixed results. This strategy has mainly been shown to support highly confident and highly interested learners in their motivation and performance, yielding negative effects on individuals with low levels of confidence and initial interest (Durik and Harackiewicz, 2007; Durik et al., 2015; Canning and Harackiewicz, 2015). Thus, telling learners about the potential usefulness of what they are learning can hinder motivational development and performance, particularly in students who doubt their abilities with regard to this content. Similarly, research on fostering utility-value beliefs with application tasks is very limited. In a qualitative interview study with undergraduate students from various disciplines (Kember et al., 2008), several participants mentioned that increasing perceived utility value in professional courses “could be accomplished by setting assignments which were authentic to the profession” (Kember et al., 2008; p. 260). Moreover, in a quantitative intervention study with high school students, participants have reported higher levels of utility value of course content after being challenged to apply course content in a simulated and authentic role-play scenario (Knogler, 2014; Knogler and Lewalter, 2014a,b). With the exception of the two studies above, we could not identify further empirical research on the application strategy in the research literature. Thus, although these strategies might be widely applied in teacher education courses, evidence of effectiveness is very scarce, and practically non-existent in the teacher education context.

Most research on fostering utility-value beliefs has focused on reflective writing exercises (Strategy 3). This strategy typically includes short writing assignments such as essays or letters in which students are tasked to reflect on how some course content relates to their lives or future careers, helping them to discover and articulate value for themselves (e.g., Hulleman and Harackiewicz, 2020). Reflective writing exercises are different from application tasks. Although both stimulate students to generate thoughts about utility value, application exercises provide specific scenarios for students to make connections, whereas reflective writing exercises require students to come up with contexts and situations which allow for making connections to course content. Findings on the effectiveness of reflective writing exercises are nuanced. Often, this intervention showed differential effects on different subgroups, for example more positive effects for students with low initial performance or low perceptions of confidence (Harackiewicz et al., 2016; Hulleman et al., 2010; Hulleman and Harackiewicz, 2009; Zeeb and Voss, 2025). Generally speaking, however, participants in laboratory and field studies have reported higher utility value, academic effort, interest, competence-related beliefs, course-taking intentions and grades after receiving this type of utility-value intervention compared to control conditions (Canning et al., 2018; Gaspard et al., 2015; Harackiewicz et al., 2016; Hulleman and Harackiewicz, 2009; Hulleman et al., 2010, 2017; Rosenzweig et al., 2020). Moreover, some studies demonstrated that direct communication of utility-value (Strategy 1) in combination with reflective writing tasks (Strategy 3) yielded positive outcomes for all learners (Canning and Harackiewicz, 2015; Gaspard et al., 2015). Taken together, of all three strategies, reflective writing tasks (Strategy 3) currently have the strongest record of effectiveness, supported by a well-established research base and consistent findings across studies (Hulleman and Harackiewicz, 2020; Lazowski and Hulleman, 2016). To our knowledge, structured, research-focused reflective writing grounded in motivational theory—unlike common formats such as learning diaries (see Molitor et al., 2025) —is not yet widely applied in teacher education. Moreover, this strategy has the advantage of being easy to implement in any course without interfering with the instructional design.

Currently, empirical research on the effectiveness of reflective writing assignments in the context of teacher education is scarce. Rochnia and Gräsel (2022), for instance, conducted a short online experiment with student teachers enrolled in Master of Education programs at German universities to test the effects of a reflective writing strategy on the self-reported utility value of educational science. Across conditions, participants read brief texts highlighting the usefulness of educational science (direct communication). In the experimental condition, participants additionally wrote a short reflection on its usefulness for classroom application. Their results showed a significant main effect (*d* = 0.15) of time, but no significant treatment-by-time interaction. That is, all participants reported more positive utility beliefs after the intervention, but the brief writing task (88 words on average) did not lead to additional gains. The authors speculated that the limited depth of engagement and the impersonal online format may have contributed to this null finding.

Subsequent findings by Zeeb and Voss (2025) lend support to this interpretation. In two experimental studies with German preservice teachers, the authors implemented brief online interventions designed to enhance either growth mindset or utility value beliefs, or both. Their utility value intervention prompted participants to actively reflect on the relevance of educational research for their future professional practice through structured elaboration tasks. Results showed significant increases in utility value and willingness to engage with research, particularly among students with lower prior competence beliefs. In a second study, they also demonstrated that a combined intervention targeting both growth mindset and utility value achieved comparable effects with high efficiency. Their success, despite the online format, underscores the importance of thoughtful intervention design, sufficient dosage, and meaningful cognitive engagement. Our study builds on and aims to extend these insights by investigating the effectiveness and added value of reflective writing assignments implemented repeatedly and authentically within a semester-long university course.

### The present study

The overall aim of the present study is to contribute to effective course design for fostering positive beliefs toward research evidence in teacher education. By drawing on expectancy-value theory and on recent research on utility-value interventions, we identified utility-value beliefs as critical beliefs in pre-service teachers and highlighted three different strategies to promote them: direct communication, application and reflective writing. Since teacher educators at university often endorse evidence-based practice in teaching (e.g., Diery et al., 2020, 2021), we assume that many of them aim—at least indirectly—to convey the relevance of educational research in their courses. Moreover, typical course designs in current teacher education programs include application tasks as default design features (Bauer and Prenzel, 2012). The strategy of reflective writing assignments, which has a strong record of effectiveness and are brief and easy to implement, is neither regularly applied in teacher education nor empirically investigated in this context. Thus, to determine the added value of this strategy we tested a *default course design* including direct communication of utility value and application tasks against an *enhanced course design* which additionally included reflective writing tasks in a pre-registered field experiment (AsPredicted.org: # 29077). In case our results indicate a higher effectiveness of the enhanced design, teacher educators might consider implementing reflective writing tasks in their courses in addition to default strategies in order to optimally promote utility value perceptions of educational research in pre-service teachers.

The strategies to promote utility value were implemented consecutively in a regular semester-long teacher education course at university and our longitudinal design included measurement probes after the implementation of each strategy as well as measurements for pre-post comparisons. Although utility-value interventions primarily target learners' perception of utility value, recent findings demonstrated that instigated psychological processes might also affect other variables. We therefore expected strongest effects on perceptions of utility value, but also assumed that other beliefs such as competence-beliefs, interest and intentions would be positively affected. Taken together, our study allowed to track students' perceptions of utility value and related beliefs in response to the implementation of different strategies across the course and to determine the impact of adding reflective writing through experimental manipulation. We posed the following research questions and hypotheses to guide our research:

### Research question and hypotheses

RQ: to what extent do the two course designs (with and without reflective writing assignments) foster participants' beliefs toward evidence from educational research?

H1: participants overall hold higher perceptions of utility value of educational research evidence after the courses (both designs) than they did before, demonstrating a main effect of time.

H2: participants in the course design that additionally include reflective writing assignments demonstrate a steeper increase of utility-value perception levels as compared to the default design.

H3: both course designs benefit participants' interest in research findings, their competence beliefs in dealing with educational research evidence and their intentions to utilize research findings in their future careers.

H4: participants in the course design that additionally includes reflective writing assignments demonstrate a steeper increase of their levels of interest in research findings, their competence beliefs in dealing with educational evidence and their intentions to utilize research findings in their future careers as compared to the default course design.

## Method

### Sample

Participants were *N* = 61 third-semester pre-service teachers at a German university, all enrolled in a required semester-long course on teaching effectiveness as part of their bachelor's degree in secondary school teacher education. The course is mandatory for pre-service teachers in this program, and students had no choice in enrolling, as no alternative courses on different (e.g., less research-oriented) topics were offered. The experiment was conducted as outlined in the pre-registration, using two groups from two consecutive fall semesters. However, course enrollment was lower than anticipated, resulting in *n* = 30 participants in one semester and *n* = 31 in the following semester. We examined potential differences between the groups across all relevant variables, and no significant differences emerged. The sample comprised *n* = 30 women (49.2%) and *n* = 30 men (49.2%), with an average age of 21.03 years (SD = 2.80). One participant did not provide information on gender. All participants were bachelor students enrolled in a secondary school teacher education program, taking courses in two (STEM) subjects, along with courses in educational science and subject didactics. The ethical commission of the German Psychological Society (DGPs) approved the study, and students gave informed consent before participation.

### Procedure and experimental design

In this preregistered study (AsPredicted.org: # 29077), we implemented utility-value intervention strategies in a double-blind randomized experiment during a regular semester-long course (14 sessions including one introduction and one closing session). The course is organized in three units: (1) Introduction to basics of empirical research on effective teaching (3 sessions), (2) Introduction to generic dimensions of teaching quality (see Praetorius et al., 2018) and effective teaching strategies for secondary education (see Knogler et al., 2022) (5 sessions), and (3) Lesson preparation and mock-up lessons, in which participants try out lesson designs based on educational research evidence with other participants serving as “students” (4 sessions). Whereas the first two units consist of lectures, group work and discussions lead by the course instructor, the third unit is self-organized by the students. Students receive three ECTS credit points for the course and are required to participate, complete individual written assignments and prepare and implement a mock-up lesson in group.

**Figure 1:**
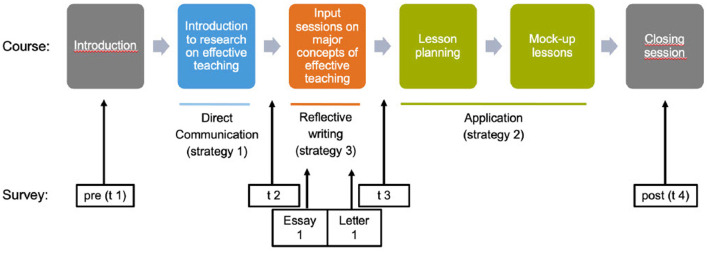
Flowchart depicting a course sequence with six phases: Introduction, Introduction to Research on Effective Teaching, Input Sessions on Major Concepts, Lesson Planning, Mock-up Lessons, and Closing Session. Surveillances, labeled pre (t1) and post (t4), occur during Introduction and during Closing Session. Strategies include Direct Communication (Strategy 1) before to t2, Reflective Writing (Strategy 3) aligned with Essay 1, Letter 1, before t3, and Application (Strategy 2) during Lesson Planning & Mock-up Lessons.

The implementation of the utility-value intervention was closely aligned with the structure of the course, which was organized into three units. In the first unit, the course instructor (second author) introduced concepts, methods, and empirical findings from educational research on effective teaching and explicitly emphasized their practical relevance for classroom instruction through presentations and discussions (direct communication). The second unit focused on key dimensions of instructional quality (e.g., classroom management, cognitive activation) and evidence-based teaching strategies (e.g., inquiry-based learning, flipped classroom). During this unit, all students completed two writing assignments—an essay and a letter—based on course content. The experimental manipulation took place within these writing tasks. Students in the control group were instructed to summarize course content in both assignments. Students in the experimental group, by contrast, were asked to summarize the material and additionally explain why the educational research-based information is useful for their (future) teaching practice. The assignments were adapted from validated utility-value intervention designs (Canning and Harackiewicz, 2015; Harackiewicz et al., 2016; Canning et al., 2018; Hulleman and Harackiewicz, 2020). Each text was approximately 600 words long and submitted as homework prior to the next session. The first assignment was an academic-style essay in which students reflected on how specific course content—particularly insights from educational research—is relevant to effective teaching and their professional development. The second was a letter addressed to an in-service teacher, in which students referred to different course content and explained why engaging with educational research is meaningful and worthwhile. The two formats were intentionally chosen to foster different forms of reflection: the essay emphasized analytical elaboration and conceptual understanding, while the letter encouraged motivational framing and perspective-taking. Together, they were designed to promote both cognitive and affective engagement with the value of educational research. Participants were randomly assigned to one of the two conditions, and received instructions via email from the first author, who was not involved in teaching the course. This procedure ensured that both the students and the instructor remained blind to the group assignments. In the third unit, all students planned and conducted mock-up lessons, with the explicit requirement to base their instructional design on relevant educational research findings (application). An overview of the study schedule is provided in Figure 1. The control condition was deliberately designed to avoid structured engagement with the utility value of research evidence. Although students in this group participated in group work and discussions to consolidate their understanding of course content, they did not receive targeted prompts or activities aimed at connecting the material to their own goals or teaching practice. While incidental reflections may have occurred, the instructor did not observe or facilitate them, and there is no indication that they played a substantial role in the control condition.

### Measures

We used adapted versions from existing multiple-item self-report scales to assess participants‘levels of perceived personal utility value (4 items, from Hulleman and Harackiewicz, 2009, e.g., “*Findings from research on effective teaching are important for my future job*.”) and general job utility value (8 items, from Zeuch and Souvignier, 2015, e.g., “*When planning support measures for students, teachers should consider scientific evidence from teaching research*.”). The internal consistency of the two scales was assessed using McDonald's omega, which yielded values between ω_range_ = 0.78–0.91 (personal) and ω_range_ = 0.71–0.83 (general) across the four measurement occasions., Since interventions targeting utility value sometimes impact other beliefs, we also measured participants' competence beliefs on applying research evidence to practical challenges (4 items, ω_range_ = 0.81–0.87, based on Georgiou et al., 2020, e.g., “*I believe that I have the necessary skills to apply knowledge of teaching research in my teaching practice*.”), participants' interest in educational research (5 items, ω_range_ = 0.83–0.90, from Wenglein et al., 2018, e.g., “*I enjoy learning about research on effective teaching”*) and participants' behavioral intentions to implement findings from educational research in their future practice (4 items, ω_range_ = 0.77–0.89, from Lenski et al., 2019, e.g., “*If I plan lessons in the future, I will take into account the findings of teaching research*.”). We asked students to rate each item on a 6-point Likert scale. Data for all variables were collected at four measurement occasions: (T1) at the beginning of the course (T2) after the first course unit (T3) after the second course unit (T4) after the third course unit/at the end of the course.

### Manipulation check

Further, we evaluated participants' articulated utility value by quantifying the extent to which their written assignments reflected the perceived usefulness of educational evidence. Recent research has demonstrated that utility-value interventions—particularly reflective writing tasks—are more effective when participants closely adhere to instructions, such as by making personal connections to the content (e.g., Backfisch et al., 2024). Drawing on established coding procedures from prior work (Canning et al., 2018; Harackiewicz et al., 2016), we assessed articulated utility value using a 4-point scale that captured the depth and specificity of utility connections in participants' texts. A score of “0” indicated no utility connection, “1” reflected utility articulated for teachers in general, “2” captured personal utility connections expressed in a general manner, and “3” denoted specific personal utility connections, such as those involving concrete scenarios. The coding process demonstrated strong interrater reliability: two independent coders assigned identical scores to 89% of the essays, and all discrepancies were resolved through discussion. The resulting weighted kappa coefficient was κ = 0.78, indicating substantial agreement between raters (Landis and Koch, 1977).

### Analytic strategy

To examine the research question and related hypotheses concerning the intervention, we used a repeated-measures MANOVA followed by separate repeated-measures ANOVAs for each dependent variable. First, independent *t*-tests were performed to test for baseline equivalence between the two groups (default vs. enhanced course design) at the first measurement occasion (T1/Pre). Next, we conducted a two-way repeated-measures MANOVA with time (T1/Pre, T2, T3, T4/Post) as the within-subjects factor and group (default course design vs. enhanced course design) as the between-subjects factor. This analysis examined the main effect of time and the time × group interaction which tests whether the pattern of change differs between groups. Partial eta-squared η^2^ was calculated as a measure of effect size.

Given a significant multivariate effect, follow-up repeated-measures ANOVAs were conducted separately for each dependent variable to further investigate the main effect of time and the time × group interaction, with *p*-values adjusted using the Holm-Bonferroni correction. To account for potential limitations in statistical power, supplementary paired *t*-tests were conducted on individual outcome variables to enhance sensitivity in detecting specific within-group changes over time. The Holm-Bonferroni correction was applied to adjust for multiple comparisons and control the family-wise error rate by adjusting *p*-values in a stepwise manner. Cohen's *d* was computed for both within-group and between-group effect sizes. All analyses were conducted using SPSS (Version 27).

In line with our pre-registration, our primary analytical approach—MANOVA and repeated-measures ANOVAs—remained consistent. However, several deviations from the pre-registered plan were made to address methodological concerns. First, we excluded course achievement as a dependent variable due to low internal consistency (Cronbach's α < 0.60) and minimal participant engagement, as reflected in short completion times and feedback indicating test fatigue. Exploratory analyses revealed no meaningful intervention effects, and including this unreliable measure would have introduced noise to the study's focus on motivational outcomes. Additionally, although we initially planned to control for covariates such as gender and high school GPA, including them did not substantively alter the results or improve model fit. Given our limited sample size, adding predictors would have increased model complexity and reduced statistical power, leading us to omit these covariates from the final model. Finally, to enhance interpretability, we reported paired-sample *t*-tests for within-group changes, in addition to the pre-registered analyses. These adjustments were made to better align the analysis with data quality and research questions while maintaining the integrity of our pre-registered hypotheses.

## Results

### Manipulation check

As expected, students in the experimental condition made higher-quality utility value connections (i.e., made more personal and more specific connections to curricular content) in their written texts (*M* = 1.92, *SD* = 0.89) than those in the control condition (*M* = 0.91, *SD* = 0.48), *t*_(46.41)_ = 5.54 *p* < 0.001. Indeed, 88% of writing assignments in the control condition did not include a personal utility value connection, in contrast to just 30% in the experimental condition. This important manipulation check indicates that the utility reflective writing intervention was successful in encouraging students to make personal connections with the course material in their writing assignments.

### Descriptive statistics

**Figure 2:**
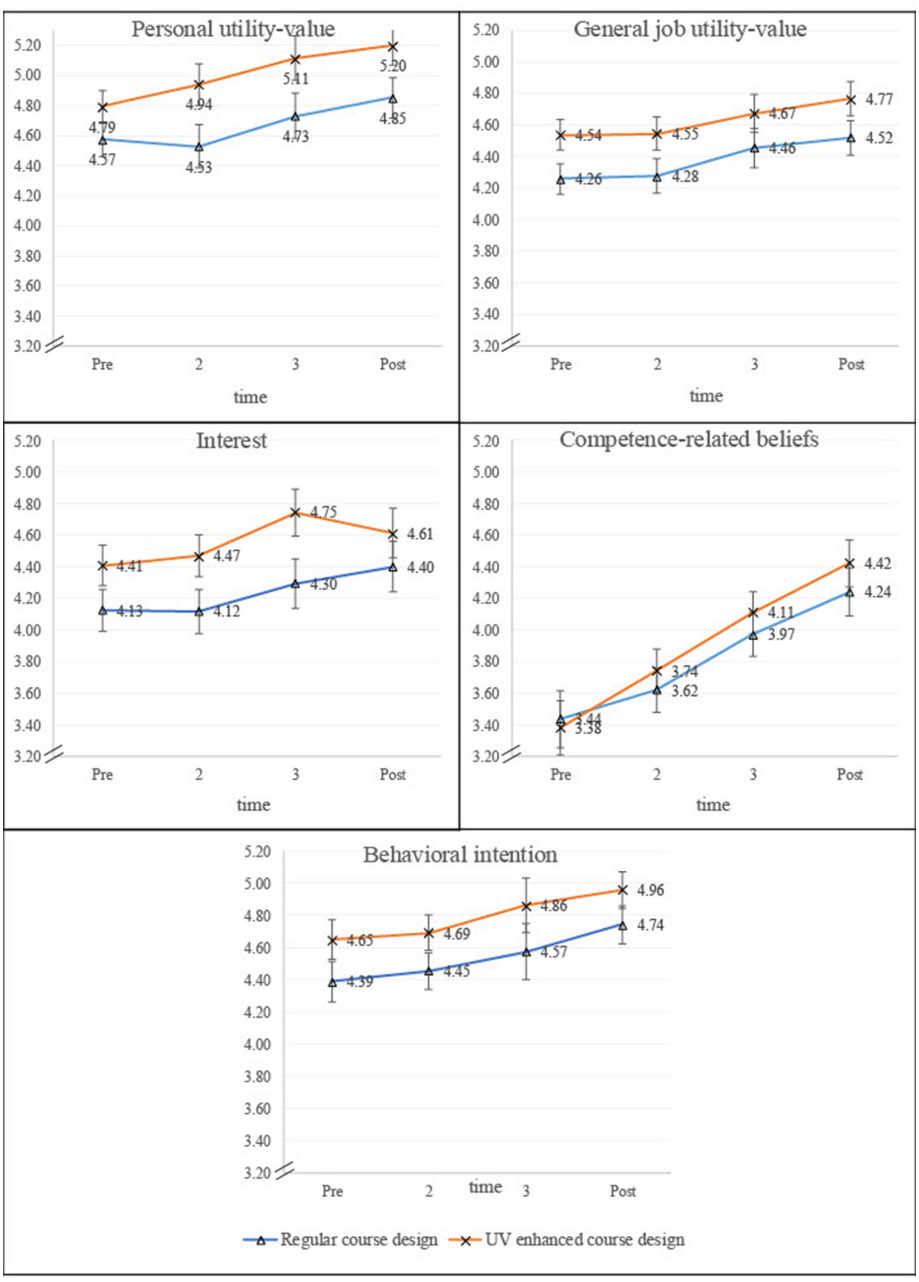
Line graphs compare “Regular course design” and “UV enhanced course design” across different factors: personal utility-value, general job utility-value, interest, competence-related beliefs, and behavioral intention. Each graph connects mean values over four time points: Pre (T1), T2, T3, and Post (T4).

Descriptive statistics for all investigated variables are presented in Table 1 for the full sample and in Table 2 separately for each condition. Correlations between all measures are provided in Table 3. Figure 2 illustrates the mean values of each investigated variable across the four measurement points. At baseline (T1/Pre), students reported relatively high utility-value ratings for educational research evidence (*M* = 4.69, *SD* = 0.58, range = 3.75–6.00) and general job utility-value (*M* = 4.39, *SD* = 0.52, range = 3.38–5.50). In contrast, competence-related beliefs regarding the application of educational research evidence to teaching practice received the lowest ratings (*M* = 3.39, *SD* = 0.89, range = 1.50–6.00).

**Table 1:** Means and standard deviations of outcome measures across the full sample (T1–T4).

	**T1**		**T2**		**T3**		**T4**	
**Variables**	***M***	***SD***	***M***	***SD***	***M***	***SD***	***M***	***SD***
Personal utility-value	4.69	0.58	4.78	0.76	4.92	0.81	5.01	0.70
General job utility-value	4.39	0.52	4.43	0.56	4.54	0.63	4.63	0.57
Interest	4.28	0.69	4.31	0.76	4.54	0.81	4.51	0.81
Competence-related beliefs	3.39	0.89	3.73	0.74	4.06	0.71	4.32	0.77
Behavioral intentions	4.53	0.66	4.61	0.58	4.73	0.89	4.84	0.65

**Table 2:** Means and standard deviations of outcome measures by course design (T1–T4).

	**Default Course Design (*****n*** = **29)**								**Enhanced course design (*****n*** = **32)**							
**Variables**	**T1**		**T2**		**T3**		**T4**		**T1**		**T2**		**T3**		**T4**	
	***M***	***SD***	***M***	***SD***	***M***	***SD***	***M***	***SD***	***M***	***SD***	***M***	***SD***	***M***	***SD***	***M***	***SD***
Personal utility-value	4.57	0.56	4.53	0.92	4.73	0.95	4.85	0.82	4.79	0.58	4.94	0.52	5.11	0.61	5.20	0.57
General job utility-value	4.26	0.51	4.28	0.68	4.46	0.79	4.52	0.66	4.54	0.51	4.55	0.40	4.67	0.48	4.77	0.46
Interest	4.13	0.67	4.12	0.81	4.30	0.93	4.40	0.84	4.41	0.72	4.47	0.63	4.75	0.68	4.61	0.84
Competence-related beliefs	3.44	0.89	3.62	0.62	3.97	0.70	4.24	0.80	3.38	0.95	3.74	0.84	4.11	0.72	4.42	0.77
Behavioral intention	4.39	0.66	4.45	0.66	4.57	0.86	4.74	0.70	4.65	0.66	4.69	0.52	4.86	0.95	4.96	0.56

**Table 3:** Bivariate Correlations between Study Variables at Pre and Post Measurement.

**Variables**	**Time**	**1**	**2**	**3**	**4**	**5**	**6**	**7**	**8**	**9**
1. Personal UV	T1	-								
2. General Job UV	T1	0.59^**^								
3. Interest	T1	0.51^**^	0.40^**^							
4. Competence beliefs	T1	0.28^*^	0.24	0.32^*^						
5. Behavioral intentions	T1	0.63^**^	0.61^**^	0.61^**^	0.26^*^					
6. Personal UV	T4	0.52^**^	0.41^**^	0.40^**^	0.21	0.44^**^				
7. General Job UV	T4	0.41^**^	0.43^**^	0.36^**^	0.07	0.46^**^	0.70^**^			
8. Interest	T4	0.49^**^	0.31^*^	0.67^**^	0.29^*^	0.48^**^	0.58^**^	0.58^**^		
9. Competence beliefs	T4	0.15	0.16	0.25	0.29^*^	0.15	0.28^*^	0.48^**^	0.34^**^	
10. Behavioral intentions	T4	0.39^**^	0.32^*^	0.47^**^	0.16	0.50^**^	0.66^**^	0.81^**^	0.63^**^	0.56^**^

^*^p < 0.05.

^**^p < 0.01.

### Baseline differences

Independent *t*-tests confirmed that there were no significant baseline differences between the two course groups at pretest across all investigated variables. No statistically significant differences were found for personal utility-value, *t*_(59)_ = 1.10, *p* = 0.276, general job utility-value, *t*_(59)_ = 1.64, *p* = 0.107, interest, *t*_(59)_ = 1.38, *p* = 0.172, competence-related beliefs, *t*_(59)_ = 0.17, *p* = 0.866, or behavioral intention, *t*_(59)_ = 1.36, *p* = 0.181. These results indicate that the two groups were comparable at the start of the intervention.

### Testing differences in beliefs across time and across course designs

The repeated-measures MANOVA revealed a significant multivariate main effect of time across all variables, V = 0.65, *F*_(15,40)_ = 4.88, *p* < 0.001, partial η^2^ = 0.65. This finding supports Hypothesis 1, which predicted that students would show significant improvements in their utility-value perceptions over time, and Hypothesis 3, which anticipated significant changes in students' interest in research findings, competence beliefs, and behavioral intentions. However, neither the multivariate group effect, V = 0.09, *F*_(5,50)_ = 1.04, *p* = 0.405, partial η^2^ = 0.09, nor the interaction effect between time and group, V = 0.10, *F*_(15,40)_ = 0.29, p = 0.994, partial η^2^ = 0.10, were significant. These results indicate that while significant improvements occurred over time, the rate of change did not significantly differ between students in the enhanced and default course designs. Consequently, Hypothesis 2, which proposed an interaction effect between time and group on utility-value perceptions, and Hypothesis 4, which predicted an interaction effect between time and group on competence beliefs, interest, and behavioral intentions, were not supported.

To further investigate the main effect of time, separate repeated-measures ANOVAs were conducted for each dependent variable. Significant main effects of time were observed for personal utility-value, *F*_(3,162)_ = 5.67, *p* = 0.001, partial η^2^ = 0.10, general job utility-value, *F*_(3,162)_ = 4.99, *p* = 0.002, partial η^2^ = 0.09, interest, *F*_(3,162)_ = 4.92, *p* = 0.003, partial η^2^ = 0.08, competence-related beliefs, *F*_(3,162)_ = 26.39, *p* < 0.001, partial η^2^ = 0.33, and behavioral intention, *F*_(3,162)_ = 5.06, *p* = 0.002, partial η^2^ = 0.09. These findings support Hypothesis 1 and 3Fi, which predicted significant improvements over time in personal utility-value, job utility-value, interest, competence beliefs, and behavioral intention. However, and consistent with the results of the MANOVA, no significant time × group interaction effects were found for any of the investigated variables, all Fs < 1. This suggests that while students improved significantly across all investigated variables over time, these improvements did not differ between the two course conditions, failing to support Hypothesis 2 and Hypothesis 4, which proposed interaction effects between time and group for specific variables (utility-value perceptions and competence-related beliefs, interest, and behavioral intentions). Figure 2 illustrates the similar developmental trajectories of both groups, further confirming the absence of a significant interaction effect.

Given the relatively small sample size, the MANOVA yielded low statistical power (approximately 30% for detecting a medium effect and 5% for detecting a small effect). To enhance sensitivity in detecting specific changes over time, additional paired *t*-tests were conducted across the full sample and separately for each course group. Analyses across the full sample indicated significant pre-post improvements. The strongest increase was observed in competence-related beliefs, *t*_(60)_ = −7.31, *p* < 0.001, *d* = 0.94. Significant improvements were also found for personal utility-value, *t*_(60)_ = −4.01, *p* = 0.001, *d* = 0.51, general job utility-value, *t*_(60)_ = −3.20, *p* = 0.011, *d* = 0.41, interest, *t*_(60)_ = −2.91, *p* = 0.025, *d* = 0.37, and behavioral intentions, *t*_(60)_ = −3.62, *p* = 0.003, *d* = 0.46. When analyzing the course groups separately, different patterns of change emerged. In the default course design, a significant improvement was observed only for competence-related beliefs, *t*_(28)_ = −3.81, *p* = 0.005, *d* = 0.71. In contrast, in the enhanced course, significant improvements were found for personal utility-value, *t*_(31)_ = −3.34, *p* = 0.010, *d* = 0.59, general job utility-value, *t*_(31)_ = −3.05, *p* = 0.025, *d* = 0.54, and competence-related beliefs, *t*_(31)_ = −6.98, *p* < 0.001, *d* = 1.23. These supplementary findings suggest that while the overall MANOVA and ANOVAs did not detect significant time × group interactions, within-group comparisons indicate that students in the enhanced course showed greater improvements in several domains, providing partial support for Hypothesis 2 and Hypothesis 4.

Taken together, the results indicate significant improvements over time across all investigated variables, confirming Hypothesis 1 and Hypothesis 3. However, no significant differences in change trajectories between course groups were observed in the primary analyses, leading to the rejection of Hypothesis 2 and Hypothesis 4. The supplementary paired *t*-tests suggest that students in the enhanced course exhibited greater improvements in utility-value perceptions and competence beliefs compared to those in the default course, highlighting potential group-specific effects that may not have been fully captured in the main analyses.

## Discussion

We started out from the observation that (pre-service) teachers rarely make use of objective sources of knowledge from educational research when confronted with practical challenges (e.g., Brown and Rogers, 2015; Dagenais et al., 2012; Franke and Wecker, 2019; Ferguson and Bråten, 2022; Patry, 2019; Thomm et al., 2021c). This deficit in evidence-based practice is noteworthy, as educational research has accumulated a rich body of theories and evidence to draw upon in classroom teaching and making use of that knowledge has shown to make a difference for student learning (Knogler et al., 2022; Bransford et al., 2000). One plausible reason for this deficit is that pre-service teachers often hold unfavorable beliefs about the utility of educational evidence for successful classroom teaching (van Schaik et al., 2018). Given that initial teacher education is a formative context for shaping such beliefs, it is important to harness the potential of university courses to support pre-service teachers in perceiving the value of theories and findings from educational research. Since knowledge on how to optimally promote value perceptions during regular teacher education courses currently seems lacking, the aim of our study was to contribute to effective course design for fostering positive beliefs toward research evidence. To this end, we tested a *default course design* including direct communication and application tasks as typically applied strategies to foster utility value against an *enhanced course design* which additionally included reflective writing tasks. Reflective writing assignments which stimulate learners to reflect on the utility-value of what they are learning have a strong record of effectiveness and are relatively easy to implement (e.g., Hulleman and Harackiewicz, 2020; Lazowski and Hulleman, 2016). As our results partially confirmed our expectations concerning the added value of reflective writing assignments, teacher educators might consider implementing them in their courses.

Since both course designs included strategies to foster utility value, we expected that pre-service teachers in both conditions would hold higher perceptions of utility-value after the courses than they did before (Hypothesis 1), with steeper gains in the enhanced condition (Hypothesis 2). Given that strategies aimed at fostering utility value have shown to affect other motivational variables, we assumed that both course designs benefit participants' interest in research findings, their competence beliefs in dealing with educational evidence and their intentions to utilize research findings in their future careers (Hypothesis 3), with greater gains in the enhanced design (Hypothesis 4).

In support of Hypothesis 1, participants across both conditions demonstrated significant increases in utility value perceptions. On average, both their ratings of general and personal utility value of educational research evidence increased over the course of the semester, even though baseline levels were well above the numerical scale mean. These results as indicated by pre-post tests and repeated measures MANOVA suggest that implementing strategies to support can help pre-service teachers to find more value in educational research. Our findings align with recent work showing that pre-service teachers report increased utility perceptions following targeted interventions (Rochnia and Gräsel, 2022; Zeeb and Voss, 2025). Notably, Zeeb and Voss (2025) demonstrated that even brief, structured online interventions—including elaboration tasks—can foster utility value and willingness to engage with research. These effects persisted at a 2-week follow-up, indicating short-term durability even with low-intensity interventions (Zeeb and Voss, 2025). The observed effect size for personal utility value (*d* = 0.51) in our study appears reasonable, especially compared to the smaller effect (*d* = 0.15) reported by Rochnia and Gräsel (2022). However, because our control group also received strategies to foster utility value, we cannot fully isolate the effects of reflective writing alone.

Regarding Hypothesis 2, results were mixed. Separate *t*-tests showed that only the enhanced course group exhibited a statistically significant gain in utility value, suggesting a possible added benefit of reflective writing. Our analysis of the written responses confirmed that students in the experimental group indeed engaged with utility-value content, supporting the validity of the intervention. These findings align with earlier research on reflective writing's impact on utility value (e.g., Shin et al., 2019; Gaspard et al., 2015; Hulleman et al., 2010). However, the interaction effect in the repeated-measures MANOVA was not significant. This null result might be attributable to ceiling effects, as participants started with relatively high utility beliefs, leaving limited room for growth. Alternatively, the small sample size may have reduced our ability to detect interaction effects. Still, these findings reflect a general trend toward improved utility perceptions in the enhanced condition, which is consistent with results from Zeeb and Voss (2025), who also found modest but meaningful changes in beliefs in short-term interventions.

In support of Hypothesis 3, participants in both groups showed gains in interest, competence beliefs, and behavioral intentions. The strongest effect was observed for competence beliefs (*d* = 0.94), suggesting that participants felt more capable of interpreting and applying educational research after the course. This finding is important given the relatively low baseline levels in this domain, which highlight the need for interventions targeting pre-service teachers' research-related self-efficacy (Thomm et al., 2021c). As indicated by previous research (Brisson et al., 2017; Canning and Harackiewicz, 2015; Hulleman et al., 2017), providing a course which explicitly supports the perception of utility value of what is taught might have affected pre-service teachers' competence related beliefs since an increased belief in the utility of course content is positively associated with course engagement and in turn with performance and perceived competence. Additionally, it is important to note that the observed gains in competence could also stem from the specific knowledge and skills participants acquired throughout the course. The hands-on experience and practical application of research findings may have directly enhanced their sense of competence in using evidence in their future teaching practices. In light of recent findings by Zeeb and Voss (2025), it is noteworthy that their study found particularly strong effects for learners with initially low competence beliefs—suggesting a compensatory mechanism that may also have operated in our sample.

Moreover, we observed a substantial increase in participants behavioral intentions to implement findings from educational research in the whole sample. Since behavioral intentions are conceived as a strong predictor for actual behavior (Fishbein and Ajzen, 2010), promoting behavioral intentions is crucial for strengthening evidence-based practice in education (Greisel et al., 2022). Interestingly, bivariate correlations in our study indicated strong associations between participants' perceptions of utility value and their behavioral intentions. This is in line with research showing that promoting the perception of utility value supports related behavioral choices, such as course taking and retention (e.g., Canning et al., 2018). A close link between perceptions of utility-value and behavioral intentions might offer some indication that utility value is a promising leverage for changing intentions and behaviors related to evidence-based practice. However, given the design of our study we cannot infer that changes in variables such as behavioral intentions or perceived competence are causally related to our intervention targeting utility value as we cannot exclude the impact of other factors related to course instruction and experience. Nevertheless, we believe that the empirically validated gains in variables which are all potential predictors of behaviors related to evidence-based practice are promising and important to consider for future research in this field.

With respect to Hypothesis 4, the results did not support our assumption. Participants in both groups showed similar change patterns with regard to self-reported competence-related beliefs, interest and behavioral intentions. Thus, according to these results, the additional implementation of reflective writing assignments as part of the enhanced course design did not show any measurable advantage compared to the default design with regard to these variables. Looking at previous utility value intervention research in other contexts, this result is not an exception. Often, the implementation of reflective writing assignments did not affect all leaners on these outcomes but only specific subgroups of learners such as students with a history of poor performance, low expectations of success (Hulleman and Harackiewicz, 2009; Harackiewicz et al., 2016; Canning and Harackiewicz, 2015) or underrepresented racial/ethnic minority students (Harackiewicz et al., 2016). At the same time, there is research which shows that utility-value interventions may affect all learners (e.g., Asher et al., 2023; Rosenzweig et al., 2020; Hulleman et al., 2017) and this is what made us formulate our hypothesis. A critical point to consider in this regard are the characteristics of the default design in our study which served as a control condition. To demonstrate effectiveness, the reflective writing assignments had to pass a relatively hard test against a “default” design which already included two strategies promoting utility value. Thus, although we do observe positive changes on all variables in the enhanced design, change scores are not significantly better than in the default design group. And although there are studies that demonstrate the superior effectiveness of combined or higher dosed interventions (Canning and Harackiewicz, 2015; Priniski et al., 2019), these studies mostly use zero utility value intervention control groups. Moreover, while the utility-value intervention in our study was consistent with the dosage used in previous research (e.g., Hulleman and Harackiewicz, 2020), it is important to acknowledge that it represents a relatively small component within a semester-long course consisting of 14 sessions. Given the larger scope of the course, the intervention's limited duration and intensity may be a factor in the non-significant group differences observed, highlighting the potential need for more extensive or intensive interventions in future studies.

### Limitations and future directions

As with any empirical work, this study has several limitations which warrant discussion. First, in our measures we used a rather narrow, one-dimensional conception of research evidence with a focus on empirical educational research. This conception is limited as researchers have put forward more differentiated conceptions of evidence when it comes to teaching (see Franke and Wecker, 2019; Kiemer and Kollar, 2021; Siegel and Daumiller, 2021; Renkl, 2022). For example, evidence may have been generated in different scientific knowledge domains, all of which are related to successful teaching. In accordance with Shulman's differentiation of three domains (Shulman, 1987), Voss (2022), for example, used a three-dimensional measurement and investigated utility-beliefs regarding evidence from education science, from subject disciplines and from subject didactics. Moreover, researchers have distinguished different forms of both scientific and non-scientific information and between “theoretical” information and “empirical” information and thus recommended to distinguish between beliefs about the utility of four different kinds of information: (a) scientific (i.e., educational) theories, (b) scientific (i.e., educational) evidence, (c) subjective theories, and (d) anecdotal experience (Franke and Wecker, 2019; Kiemer and Kollar, 2021). Since our course was mainly focused on learning about empirical educational research, we chose a corresponding focus in measurement. Future research in teacher education might broaden this perspective and offer more nuanced insights.

Second, a limitation of our research design is the lack of a zero utility-value intervention control group. This design only allowed us to test the effectiveness of reflective writing assignments in addition to the effects of two other strategies embedded in the curriculum. While previous research often uses zero-intervention control groups to isolate the effects of specific strategies, our control group reflected real-world curricular conditions, strengthening the study's internal validity (see Harackiewicz and Priniski, 2018). However, we acknowledge that the absence of a zero-intervention control group limits the ability to isolate the effect of the reflective writing intervention. Future studies in teacher education, including those in controlled lab settings, could benefit from designs that isolate and test single strategies against a zero-intervention control group.

Third, another limitation of this research is the lack of statistical power due to the relatively small sample size. This limitation may have hindered our ability to detect subtle effects, such as the interaction between group and time. The low power raises concerns about the reliability of the non-significant interaction effect, and a larger sample would improve the ability to detect such interactions. Additionally, previous research has shown that certain subgroups, such as students with low initial performance or low confidence, tend to benefit more from utility-value interventions (Harackiewicz et al., 2016; Hulleman et al., 2010; Hulleman and Harackiewicz, 2009). However, our small sample size limits the detection of these subgroup differences. Larger and more diverse samples are needed to effectively test these effects and improve the generalizability of findings.

Finally, from a bigger picture perspective, barriers to evidence-based practice are located on different levels and thus need to be addressed on different levels to optimally support evidence-based practice in teaching (van Schaik et al., 2018). Due to its targeted approach and its clear focus on pre-service teachers and their perception of the utility value of research evidence, this research was aimed at creating an impact on a central psychological factor on the individual level and to change important beliefs early on in the professional career of teachers. Next to the individual teacher level, research has identified barriers on at least three other levels which include the research knowledge level, the school organization level and the communication level. According to current research (van Schaik et al., 2018) barriers are still present at all four levels. Thus, our approach is limited by its focus and future efforts should also be aimed at increasing the accessibility of research knowledge for teachers (e.g., by offering selected and easy-to-access information through brokering activities see, e.g., Knogler et al., 2022; Diery et al., 2021), at promoting supportive school leadership and at promoting collaboration and reciprocal partnerships between teachers and researchers as other key facilitators. Importantly, however, progress on one level can help to support progress on another level, as for example negative beliefs seem to be related to issues of accessibility as teachers with negative beliefs are more likely to criticize research knowledge as being inaccessible and incomprehensible (Broekkamp and van Hout-Wolters, 2007).

## Conclusion

Initial teacher education at university represents a critical window for future teachers shaping their beliefs toward research evidence. In this study, we identified and tested research-based intervention strategies to foster perceptions of utility-value of educational research in pre-service teachers. Our research documents that important beliefs regarding evidence-based practice can change in relation to research-based courses at university. From our results, however, we currently cannot conclude that course designs which additionally include reflective writing assignments are more effective than course designs which only include communication and application as default strategies to promote utility-value. This suggests that perceptions of utility-value of educational research may also be effectively fostered in courses that rely on those default strategies. Nevertheless, our results also demonstrate that participants' perceptions of utility-value significantly increased following the implementation of the enhanced course design, but not in default design. Hence, this study still offers some limited support for the additional value of reflective writing assignments.

## Data Availability

The raw data supporting the conclusions of this article will be made available by the authors, without undue reservation.
